# Caregivers’ perception of the caring challenges in coronavirus crisis (COVID-19): a qualitative study

**DOI:** 10.1186/s12912-021-00607-1

**Published:** 2021-06-19

**Authors:** Fateme Mohammadi, Mojtaba Farjam, Yousef Gholampour, Mojtaba Sohrabpour, Khodayar Oshvandi, Mostafa Bijani

**Affiliations:** 1 Chronic Diseases (Home Care) Research Center and Autism Spectrum Disorders Research Center, Department of Nursing, Hamadan University of Medical Sciences, Hamadan, Iran; 2 NonCommunicable Diseases Research Center (NCDRC), Fasa University of Medical Sciences, Fasa, Iran; 3 Mother and Child Care Research Centerx, Mother and Child Care Research Center, School of Nursing and Midwifery, Department of Nursing, Hamadan University of Medical Sciences, Hamadan, Iran; 4 Clinical Research Development Unit, Valiasr Hospital, Fasa University of Medical Sciences, Fasa, Iran

**Keywords:** Caring challenges, Caregiver, Emerging diseases, Coronavirus, Qualitative research

## Abstract

**Background:**

The nurses act as the guardians of people’s health by preventing, controlling, and curing emerging diseases, including coronavirus, a highly infectious and contagious disease which has presented the caregivers in the Iranian healthcare system with many clinical challenges. In view of lack of research on the clinical challenges which arise during health crises, emerging diseases included, there is need for further investigation of those clinical challenges and dilemmas. The aim in present study was to describe the caregivers’ experiences of the caring challenges in patients with coronavirus (COVID-19).

**Methods:**

The present study is a qualitative work with a phenomenological-descriptive design. Data were collected via semi-structured, in-depth, individual interviews. The collected data were analyzed according to Colaizzi’s method. The participants were 23 members of the medical staff responsible for coronavirus patients in Iran who met the inclusion criteria. The participants were selected via purposeful sampling which was continued to the point of data saturation.

**Results:**

The results yielded 3 main themes “psychological tension”, “inefficient management”, and “contextual factors” with 11 categories.

**Conclusion:**

In dealing with coronavirus patients and providing quality care to them, nurses face various clinical challenges which affect their performance. Administrators must, alongside giving instructions to people on how to prevent the coronavirus disease and taking effective safety measures, make sure that clinical centers are managed efficiently in order for nurses to fulfill their caring objectives satisfactorily.

**Supplementary Information:**

The online version contains supplementary material available at 10.1186/s12912-021-00607-1.

## Introduction

In recent decades, medical advances in developed and a few developing countries have made it possible to cure bacterial diseases with antibiotics as well as to prevent and control viral diseases, including pertussis, measles, polio, and rubella [1, 2]. These achievements have created a wrong sense of optimism regarding the treatment of most contagious/infectious diseases which were epidemic; indeed, in many developed countries, priority is given to non-communicable diseases in medical-clinical interventions [3]. However, such phenomena as antibiotic resistance, genetic mutations, novel bacterial pathogens, as well as emergence and reemergence of bacterial conditions have raised many doubts about the comforting prospect of our relative immunity from communicable diseases [4–6]. The term “emerging” is used to describe infectious diseases which occur for the first time in the world, an area, or a population, or pathogenic infections which existed before but have become more severe or acquired drug resistance, or pathogenic infections which have become more widespread [7, 8]. Based on this definition and statistics reported by WHO, there are currently over 30 emerging infections of varying types, geographical extents, and severities in the world [9]. According to the Centers for Disease Control and Prevention (CDC), the nature of emerging viral infections is such that there is no specialized vaccine or treatment for them and they, consequently, become epidemic over wide geographical areas, infect an increasing number of people, cause many mortalities, and inflict considerable medical costs [10].

One of those emerging diseases is coronavirus (COVID-19) which started from China in 2019 and has become a pandemic in many countries, including Iran. The initial symptoms of the disease are similar to those of influenza, but the virus gradually develops and affects the respiratory, cardiac, and renal systems (http://www.jamaicaobserver.com/international/who-chief-demands-end-to-hoarding-masks-gloves_188640?profile=1470). The patients who are transferred to medical centers often show signs of dyspnea, tachypnea, and respiratory failure [11]. However, as there has not been a certain treatment or vaccine for COVID-19 up to now, accordingly, over 137 million people in the world have contracted the disease in the past 3 months, over 265,000 of whom have lost their lives as a result (http://www.jamaicaobserver.com/international/who-chief-demands-end-to-hoarding-masks-gloves_188640?profile=1470) [11].

In Iran, however, the spread of COVID 2019 has been complex. At the beginning of the pandemic, Iran had the third highest reported cases of COVID 2019, after China and Italy. Currently, Iran is faced with the fourth wave of the pandemic, while many other countries are going through the second wave [12]. Although the majority of Iranians wear masks, but inappropriate economic situation has resulted in very few restrictions on work, activity and travel in all cities of Iran; thus, large numbers of people use public transportation every day, and the streets is crowded. In addition all employees are at work every day and telecommuting from home is very low [12]. These factors have caused about 2 million people in Iran to become infected with COVID 2019, over 65,000 of whom have lost their lives [13, 14].

Nurses, due to the special nature of their occupation, are one of the most important health professionals, who have been in close contact with these patients during the COVID 2019 pandemic; nevertheless, nurses face many care challenges in caring for coronavirus (COVID-19) patients [12]. In Iran, nurses have been the most important caregivers of patients with COVID 2019 over the four waves of this pandemic and have experienced many care challenges in caring for these patients [12]. It would therefore seem necessary to investigate and specify the concept of care challenges and the factors related to it, in caring for patients with COVID 2019.

Due to the importance of this pandemic and the lack of comprehensive studies on the care challenges in caring for patients with COVID 2019 in the Iranian society, this study was conducted using a phenomenology approach aimed at describing the experiences nurses of the caring challenges in patients with COVID-19 in Iranian society.

## Methods

### Study design and research question

A qualitative study with a descriptive phenomenology approach was used in order to answer the research question’ how nurses experience and understand the caring challenges in patients with coronavirus (COVID 2019)?’ from February to May, 2020 (during the early days of COVID 2019). Descriptive phenomenology is a philosophical and a scientific method and is widely used in social science research as a method to explore and describe the lived experience of individuals. In reporting this qualitative study we have adhered to the COREQ (Consolidated criteria for reporting qualitative research) guidelines [15].

### Participants

Nurses who provided care to COVID-2019 patients were the sample of this study. The inclusion criteria were being willing to participate, being in practice in one of the hospitals assigned for Covid-19 patients, having at least 1 month of work experience in caregiving wards for patients with COVID2019, being Iranian, having a good command of Farsi, and being able to provide adequate and rich information. The corresponding author receive the phone number of these 45 nurses working in COVID 2019 wards from the office of nursing services, in three hospitals affiliated with University of Medical Sciences in the west of Iran. Twenty-five nurses who provided care to COVID-2019 patients met the inclusion criteria for this study, while 23 nurses agreed to participate in the study. In addition, 22 nurses refused to participate in the study due to intensive work shifts, fatigue, and lack of opportunities for interviews. Thus, the researcher started the study with purposive sampling method from among 23 participants and continued the interviews until data saturation was reached and no new categories emerged. Purposeful sampling is widely used in qualitative research for identifying and selecting information-rich cases related to the phenomenon of interest. Information-rich cases are those from which one can gather important information about issues of research. Studying information-rich cases yields insights and in-depth understanding rather than empirical generalizations [16].

### Data collection

Data were collected using 23 individual, semi-structured interviews. The video calls with “WhatsApp” for interviews were conducted at times which were convenient for the participants. The corresponding author conducted and analyzed all interviews. Each interview began with a few general questions, including “Can you describe a typical day of caring for coronavirus patients?”, “What are your feelings when you are caring for these patients?”, “What factors influence your performance as a caregiver?”. Subsequently, based on the respondents’ answers, follow-up questions would be asked to increase the clarity of the information—the questions included, “Can you explain further?”, “What do you mean by that?”, and “Can you give an example?”. The interviews were oriented around the main objective of the study. Each interview lasted between 45 and 75 min. The interviews were recorded with the verbal and writing consent of the participants. Immediately after each interview, the interviews were listened to by the corresponding author several times to develop a general understanding and deep insights, after which they were transcribed on paper, along with the field notes. After each interview, data analysis was carried out and the next interview was scheduled. Interviews continued until no other new sub-themes were created and data reached the saturation. The collected data were finally analyzed in MAXQDA v. 2007. (Additional file 1: Interview Guide and English language Interview).

The collected data were analyzed according to Colaizzi’s method, which consists of 7 steps:

1- Reading and re-reading each transcribed interview, 2- Extracting important terms and phrases from the transcripts, 3- Assigning meaning to the extracted units, 4- Organizing and categorizing similar units, 5- Presenting comprehensive descriptions of the extracted categories, 6- Creating a basic paradigm of the subject under study according to the extracted categories, 7- Confirming the basic paradigm by having the participants verify the themes and categories [17]. An example of an analysis is outlined in Table 1.

**Table 1:** An example of an analysis

Step1	Step 2	Step 3	Step 4	Step 5	Step 6	Step 7
Interview was recorded and transcribed	I was in the central infection ward where the coronavirus patients were, in my protective clothing and shields, gloves, and goggles which I had been wearing for 6 h on my shift. I was soaked with sweat and the spots where my face shield and glasses were pressing on my face and ears were killing me. I’d never been exposed to this much tension at work.	Annoying work clothes Much work tension	Working in difficult conditions	Stress at work	psychological tension	Member checking was done. To do this, the extracted concepts and themes were submitted to 4 participants who confirmed that the findings were in line with their understandings and interpretations.
	There were more and more patients and their conditions were getting worse and worse. I’d been in the hospital since yesterday and the sudden increase in our workload was a huge shock to me.	A lot of patients with worse conditions Sudden increase in workload	High workload			

## Rigor

Accordingly, to increase the validity and accuracy of the data, the researchers used a combination of sources including semi-structured interviews, prolonged engagement with the data, member check, and peer check. To do this, the extracted concepts and themes were submitted to 4 participants and 4 peers; They stated that the findings were in line with their understandings and interpretations. Furthermore, the researcher limited the textual reviews in order to reduce bias in collecting, analyzing, and coding of the interviews to enhance the validity of the data. Finally, confirmability was acquired through exact recording of participant narratives and detailed reporting of the study to provide the possibility of follow-up for other researchers.

## Ethical considerations

All participants gave written informed consent to participate in the study. The present study was conducted in accordance with the principles of the revised Declaration of Helsinki, a statement of ethical principles which directs physicians and other participants in medical research involving human subjects. Also, the study was approved by the local Ethics Committee of Fasa University of Medical Sciences, Fasa, Iran (IR.FUMS.REC.1398.188).

## Results

A total of 23 nurses (12 females and 11 males) within an age range of 24–52 years were interviewed. The majority participants were married (69.57%) and had a bachelor’s degree in nursing (56.53%) with a work experience range of 1–16 years. The personal characteristics of the participants are reported in Table 2.

**Table 2:** Social demographic characteristics of the participants

Participants	Sex	Marital status	Educational level	Work experience (years)
P1	Female	Single	Bachelor’s degree in nursing	3
P2	Female	Married	Bachelor’s degree in nursing	10
P3	Male	Married	Bachelor’s degree in nursing	7
P4	Female	Married	Bachelor’s degree in nursing	2
P5	Male	Married	Associate’s degree in Nursing	8
P6	Female	Single	Bachelor’s degree in nursing	7
P7	Female	Married	Bachelor’s degree in nursing	9
P8	Male	Married	Master’s degree in nursing	10
P9	Female	Single	Master’s degree in nursing	8
P10	Male	Married	Bachelor’s degree in nursing	16
P11	Female	Married	Bachelor’s degree in nursing	5
P12	Male	Single	Master’s degree in nursing	13
P13	Male	Single	Master’s degree in nursing	3
P14	Male	Married	Bachelor’s degree in nursing	1
P15	Female	Married	Master’s degree in nursing	2
P16	Male	Single	Bachelor’s degree in nursing	13
P17	Female	Married	Master’s degree in nursing	5
P18	Male	Married	Bachelor’s degree in nursing	3
P19	Female	Single	Master’s degree in nursing	7
P20	Male	Single	Bachelor’s degree in nursing	12
P21	Female	Married	Associate’s degree in Nursing	6
P22	Female	Married	Bachelor’s degree in nursing	3
P23	Male	Married	Master’s degree in nursing	9

Three main themes— psychological tension, inefficient management, and contextual factors—and 11 categories were extracted from the collected data (Table 3).

**Table 3:** Themes and categories extracted from content analysis

Theme	Category
***Psychological tension***	Stress at work Terror and anxiety Depression
***Inefficient management***	Lack of a preset action plan Lack of preparation drills Inadequate supply of high-quality services Inadequate supply of equipment and facilities Poor provision of information to the public
***Contextual factors***	The severity, type, and rate of transmission of the infection Incorrect hygiene beliefs Opportunism

### Psychological tension

The participants declared that the following inflicted considerable psychological tension on them: the sudden emergence of the disease, the fast increase in the number of the infected, deaths of young colleagues without a major illness in their medical history, extended work shifts, terror of contracting the disease or transferring the disease to one’s family, and longs hours away from one’s family. All these sources of psychological tension can influence the quality of care provided by the caregivers. The theme of invasion of psychological tension is divided into three categories: stress at work, terror and anxiety, and depression.

#### Stress at work

The nurses participating in the present study stated that the sudden emergence of the novel coronavirus in Iran and the subsequent increase in the number of suspected and confirmed cases of infection in clinical centers, the rise in their workload and extension of work shifts, having to wear protective equipment continuously, and the critical (red alert) conditions in the hospitals inflict considerable occupational-psychological tension on the personnel. This tension, in turn, threatens the quality of care.

*“I was in the central infection ward where the coronavirus patients were, in my protective clothing and shields, gloves, and goggles which I had been wearing for 6 hours on my shift. I was soaked with sweat and the spots where my face shield and glasses were pressing on my face and ears were killing me. There were more and more patients and their conditions were getting worse and worse. I’d been in the hospital since yesterday and the sudden increase in our workload was a huge shock to me. I’d never been exposed to this much tension at work. But I have to work and give care in this distressing situation. Well, such a shock and workload is a challenge to my caregiving” (Participant 5).*

#### Terror and anxiety

The participants stated that the emergence of the novel coronavirus and its high speed of transmission have caused a great deal of fear in ordinary people and even the nurses who are in close contact with the infected patients. These personnel are worried about their own contracting the disease or transferring it to their colleagues and families. Moreover, the deaths of a few members of the nurses who were responsible for coronavirus patients have struck fear into the hearts of the nurses.

*“Well, everyone is worried about getting sick. We are in direct contact with these patients, always taking their sputum samples, or suctioning the very sick patients. We are afraid that we might get the disease or give it to a colleague or to our families. Some of our colleagues have gotten sick and two of them who were young too passed away. Well, we are all naturally filled with a deep sense of fear and anxiety” (Participant 16).*

#### Depression

The participating personnel also mentioned that their constant presence in the hospitals designated for coronavirus patients, minimum contact and communication with their colleagues, and being away from their families over this period have caused them to experience signs of depression. Despite their efforts to keep their spirits, as well as the spirits of their colleagues and patients up, they still cannot avoid the psychological tension.

*“During our shifts, we try to avoid all unnecessary contact and conversation with our colleagues and patients. Everyone is very careful. But all these intensive work shifts, our need to be overly cautious, and our distance from our families have made us kind of depressed. Everyone looks anxious and sad. Of course, we sometimes play music and dance to it to keep our spirits and the patients’ spirits up and get away from this sense of depression. This kind of behavior is not acceptable to the system, but even the administrators and authorities understand our situation now” (Participant 3).*

### Inefficient management

According to the nurses participating in the present study, efficient management is a key factor in successfully handling crises, including the coronavirus crisis in Iran, and minimizing clinical challenges. Although the healthcare system in Iran has developed significantly over the past decades, there are certain deficiencies and defects in these systems which hinder effective handling of crises. This theme consists of five categories: lack of a preset action plan, lack of preparation drills, and inadequate supply of high-quality services, inadequate supply of equipment and facilities, and poor provision of information to the public.

#### Lack of a preset action plan

The nurses mentioned that planning and preparing for infection crises, including coronavirus, will minimize anxiety and fear in the public and the nurses, reduce social chaos, and improve organization in hospitals.

*“A preset action plan that determines which hospitals in every province are the primary medical centers for treating patients in case of infectious or biological diseases does not exist, or if it does, the* nurses *are not aware of it. This disease suddenly became an epidemic and they decided which hospitals should be infection care centers on an ad hoc basis. Out of nowhere, we were informed that our hospital is a coronavirus center. Well, in such conditions, everyone gets nervous and the medical staff faces challenges in providing care. The existence of a preset action plan for such situations, like predetermining the hospitals and units which should be in charge in case of an infectious disease and even pre-training the nurses, can help control these clinical challenges” (Participant 9).*

#### Lack of preparation drills

One of the important categories extracted in the present study is lack of preparation drills. The nurses declared that they had not had any drills related to infectious and biological epidemics to be at least slightly prepared for such crises. Only the medical staff of military hospitals is given drills and training to be ready for health-related crises.

*“There were never any drills for the* nursing personnel *of organizations managed by the Ministry of Health to prepare them for infection crises. There are only occasional mock drills for dealing with a crisis for the nurses and doctors at military hospitals. Well, we hadn’t been trained and this affects our handling of this crisis as well as ability to provide effective care” (Participant 18).*

#### Inadequate supply of high-quality services

The participants stated that though they were making their best efforts to provide care to the coronavirus patients, the large number of the patients who were admitted to the designated hospitals and lack of staff plus test kits made it impossible for them to provide timely and high-quality care.

*“There are too many patients in the clinics and emergency departments of the hospitals selected for coronavirus patients, we have shortage of medical staff and test kits, and the patients sometimes have to wait for some time to be tested or admitted, which makes them annoyed and aggressive whereby providing care becomes a challenge” (Participant 7).*

#### Inadequate supply of equipment and facilities

In the present study, the nurses mentioned that in order to provide high-quality care, caregivers must have access to the necessary equipment and facilities in abundance so that they can provide care with peace of mind.

*“When you enter the ward, all you get is an apron, a pair of gloves, and a mask, and throughout the shifts, they keep saying that we are short of equipment. I agree that in these conditions we need to conserve, but lack or unavailability of equipment makes caring for coronavirus patients a challenge” (Participant 21).*

#### Poor provision of information to the public

The participants stated that the awareness campaigns as well as self-care and prevention educational programs for coronavirus all started when the disease had already emerged and was becoming an epidemic. This posed a major challenge to controlling the spread of the infection and providing high-quality care.

*“It was when the coronavirus appeared and was becoming widespread that the television and social media began to educate the public. Before that, people and the* nurses *didn’t receive any information to learn about the coronavirus and how to deal with infected patients. It is true that later people were educated to some extent especially through the television, but during the first few days of the emergence of the infection, we were faced with an influx of patients who didn’t know what to do” (Participant. 1).*

### Contextual factors

Another theme extracted in the present study is contextual factors. According to the participants, the principles and manners of providing care are influenced by certain cultural and environmental factors, including the severity of an infectious disease, how it is transmitted, the public’s hygiene beliefs in every culture, and some people’s selfish attempts to capitalize on the conditions in a crisis, like coronavirus. This theme is divided into three categories: the severity and rate of transmission of the infection, incorrect hygiene beliefs, and opportunism.

#### The severity, type, and rate of transmission of the infection

The nurses declared that the little-known nature of this emerging infection (COVID-19), lack of a definite cure for it, its long incubation period, and, most importantly, its very high rate of transmission, have increased the number of people who carry the virus and the patients who visit medical centers, a major challenge to the healthcare system.

*“Unlike other infections, like H1N1, this infection is easily transmitted from person to person and the virus survives on surfaces for long periods, thus requiring systematic and quick disinfection. Well, with the sudden emergence of the infection, many healthy and infected people came to medical centers. This overcrowding of people resulted in a lot of close contact between them and the transmission of the disease. All in all, the high rate of transmission of this infection increases the number of patients and makes caregiving difficult” (Participant 6).*

#### Incorrect hygiene beliefs

Another important category of the theme of contextual factors is incorrect hygiene beliefs. The participants stated that in every culture, including Iran, people have their own beliefs about hygiene. Nevertheless, occasionally these beliefs, as in the case of coronavirus, are not based in fact and lead to additional health problems, whereby the rate of visits made to medical centers is increased, which adversely affects the quality of care.

*“It is a custom for people in Iran to shake hands and kiss. On the other hand, many people don’t observe hygiene behaviors, such as washing their hands properly after a handshake or toughing surfaces. Such behaviors put them at greater risk of catching the infection. Also, many Iranians believe in the healing powers of herbs—it is true that consumption of such herbs as cinnamon, ginger, and garlic helps with preventing colds and relieving coughs and sore throats, but overuse of them can trigger allergic reactions, especially in the elderly with a history of cardiovascular disease or diabetes. This increases the number of people who have the symptoms of a coronavirus infection in medical centers, which challenges caring for real patients” (Participant 14).*

#### Opportunism

Another danger to public health and healthcare which the participants referred to was the opportunism of some businesspeople who hoard or increase the cost of personal protective equipment, including gloves and masks, and certain foods.

*“The prices of gloves and masks suddenly tripled. In a short time, drugstores ran out of masks and gloves. The prices of food products that contain vitamin C went up, too. Even in these conditions, some people remain opportunistic and seek to make large profits. The opportunists hoarded millions of masks and gloves. Such inhuman behaviors pose a greater threat to public health in the coronavirus crisis” (Participant 14).*

## Discussion

The challenges of taking caring of coronavirus patients in the present study fall into three themes: psychological tension, inefficient management, and contextual factors. In the present study, psychological tension was a major caring challenge from the nurse’s perspective. The nurses are in the front line of care and treatment in the healthcare system when health crises, including the coronavirus epidemic, strike. Yet, in providing care, they are faced with many challenges [18, 19]. Similarly, two other studies report that caring for patients with an infectious disease inflicts unusual work overload, terror, and anxiety on caregivers, which can have an adverse effect on their performance [4, 6].

Inefficient management was another theme extracted in the present study. Correct management of crises and the existence of a preset action plan can prove very effective in controlling crises and minimizing causalities as well as financial costs. However, in the present study, the caregivers mentioned that the healthcare system lacked systematic management and preset action plans, such as predetermining hospitals as centers for infectious epidemics and organizing practice drills to train and prepare the nurses for health crises. Similarly, in their study, Nakhaei et al. introduced a category named “lack of a comprehensive health plan” and stated that there is an obvious lack of comprehensive planning for health-related crises [6]. Among the categories under the theme of inefficient management in the present study were lack of planning and prearranged measures toward equipping hospitals for high-quality care, lack of diagnostic test kits, and lack of equipment as well as facilities required for providing care to coronavirus patients. In the study of Vasli et al., the two categories of “insufficient human resources” and “ insufficient non-human resources” were presented as the causes of lack of efficient personnel in emergency care departments, work overload, and unavailability of necessary equipment in times of crisis, thus requiring more systematic management and planning [19].

Another category of this theme was poor provision of information to the public. The participants declared that they had not been provided with any education or preset action plans for handling the coronavirus and that the entire planning and decision-making began when the infection had already started to become widespread. As mentioned in many studies, timely and proper provision of information is a key factor in controlling critical conditions and minimizing causalities as well as financial costs [20]. No other studies referring to the role of official announcements in health crises and epidemics was found, which can be due to the different nature, severity, and very high rate of transmission of COVID-19 compared to other infections. The novel coronavirus has a long persistence and is transmitted very quickly, infecting a large number of people in a short time. Even though Iranian officials started to educate the public via text messages and the television with the emergence of COVID-19, it appears that the public was not informed properly enough.

“Contextual factors” constituted another theme extracted in the present study. In health crises, especially when an infectious disease becomes a pandemic, environmental and contextual factors, including the type, severity, and rate of transmission of the disease, as well as people’s values and beliefs in a culture, can have a significant impact on the health status of the people [18, 21–24].

Many Iranians move to larger cities, like Tehran, for higher incomes and better access to facilities. When COVID-19 was increased many of the people would go to their birthplaces where the air is cleaner and save themselves and their families from the coronavirus crisis in the capital. These thoughts and acts increased commutes on the roads and resulted in faster spread of the disease as well as increase in the number of patients. Similarly, other studies show that the nature and severity of an infection are important contextual factors which affect the management of health crises [18, 25]. On the other hand, Iranian’s greeting behaviors, including shaking hands, kissing, and offering their food to others in public places, along with failure to wash their hands regularly, wear gloves at work, and observe the principles of sanitation are wrong hygiene beliefs which contribute to the spread of infections. According to other studies, again the public’s hygiene beliefs and behaviors are an influential factor in maintaining and improving public health [26, 27].

Another important category extracted from the data in the present study was the opportunism of some businesspeople which has challenged the healthcare system in Iran in dealing with the coronavirus crisis. Sometimes, opportunists hoarded masks, gloves, and certain food products, thereby making it harder for others to cope with the crisis and posing additional challenges to healthcare [28, 29]. The act of hoarding food and vital equipment at the time of wars, national crises, and natural disasters is condemned all over the world. The president of WHO has warned pharmaceutical factories and producers of medical equipment about hoarding and trying to raise the prices of protective medical equipment, including masks and gloves, stating that this will contribute to the spread of the coronavirus, increase the number of casualties, and challenge healthcare systems further. This would warrant taking legal action against those who hoard medical equipment (http://www.jamaicaobserver.com/international/who-chief-demands-end-to-hoarding-masks-gloves_188640?profile=1470).

Finally, there have been various obstacles to providing high-quality care to coronavirus patients in Iran. From the perspective of the participants in the present study, in order for the nurse to provide high-quality care to the infected, the public should be educated more properly, security measures should be taken to control travel between cities, sufficient equipment and facilities should be provided, and the management of the crisis must be better organized.

### Limitations

One of the limitations of the present study was the limited access to nurses and the impossibility of conducting face-to-face interviews, which was due to the high occupancy of nurses and the high prevalence of COVID2019 in Iranian society. Other limitations included the fact that data were collected through individual interviews only — other methods of data collections could have added to the reliability of the results of this qualitative work. Accordingly, it is suggested that future studies employ, in addition to individual interviews, other ways of collecting data, including observation and field notes.

## Conclusion

Psychological tension, inefficient management, and contextual factors were the main challenges of caring for patients with COVID 2019 crisis in Iran. Health officials and policy-makers can use the findings of the present study to create a cultural, professional, and organizational context in which possible caring challenges COVID 2019 have been largely eliminated and nurses have psychological peace thereby performing more satisfactorily.

## Supplementary Information

**Additional file 1.** Interview Guide and English language Interview.

## Data Availability

The datasets used and /or analysed during the current study are available from the corresponding author on reasonable request.vailability of data and materials.
