# Supplementary material for: Caregivers’ perception of the caring challenges in coronavirus crisis (COVID-19): a qualitative study
Source: BMC Nurs. 2021 Jun 19;20:102. doi: 10.1186/s12912-021-00607-1 (PMC8214044; doi:10.1186/s12912-021-00607-1)
Supplement: Supplementary file 1 — Additional file 1. Interview Guide and English language Interview. [file 12912_2021_607_MOESM1_ESM.doc]

**Additional file2: (Interview Guide and English language Interview).**

| **Interview Guide**  Thank you for accepting to be interviewed by us. The study we are undertaking is to understand more about the Thank you for accepting to be interviewed by us. The study we are undertaking is to understand more about the “Caregivers' Perception of the Caring Challenges in Coronavirus Crisis (COVID-19): A Qualitative Study”.  I will be asking you several questions which are relevant to the study. You may respond to these queries in any way you feel comfortable. It is perfectly fine if you do not want to respond. At any point during the interview, if you are not clear about any questions, you are free to clarify the same with us and ask us to explain further. The information obtained during the interview will be kept confidential and will be shared only with the research team. We would like to audio record the interview in order to ensure that we do not miss out any salient issues. The recordings will be kept confidential. Your identity will be protected and your interview will also be labeled in codes. Is it OK with you that we audio record the interview?  **Interview questions**:  1."Can you describe a typical day of caring for coronavirus patients?  2."What are your feelings when you are caring for these patients?  3. "What factors influence your performance as a caregiver?  **English language version of interview**  Date: 20 April2020 11.21 Am Participant 5  Hello dear, thank you for your time and attention and participation in the interview.  Would you please explain about your experience about caring challenges in caring of patients with COVID 2019. Can you describe a typical day of caring for coronavirus patients?  COVID unit and caring for these patients has many caring challenges. Patients have different cultures and attitudes. Also, conditions and causes of infection are different in these patients. These patients are very sensitive to our looks, behaviors and speech. On the other hand, it is very difficult to work in the COVID ward with alot of patients. When we work with these patients, we must behave in such a way that we do not get sick. We face many care challenges.  Please explain more? What does ‘caring challenges in caring of these patients?  "I was in the central infection ward where the coronavirus patients were, in my protective clothing and shields, gloves, and goggles which I had been wearing for 6 hours on my shift. I was soaked with sweat and the spots where my face shield and glasses were pressing on my face and ears were killing me. There were more and more patients and their conditions were getting worse and worse. I'd been in the hospital since yesterday and the sudden increase in our workload was a huge shock to me. I'd never been exposed to this much tension at work. But I have to work and give care in this distressing situation. Well, such a shock and workload is a challenge to my caregiving.  Can you give others factors influence your performance as a caregiver?  Intensive work shifts, our having to be overly cautious, and our distance from our families have made us kind of depressed. |
| --- |
